# Research potential and mechanism of rare earth elements promoting cadmium remediation efficiency in hyperaccumulator plants

**DOI:** 10.3389/fpls.2025.1749732

**Published:** 2026-01-09

**Authors:** An Shi, Wenhao Yang, Beibei Wang, Wen Zhang

**Affiliations:** 1 Hainan Key Laboratory of Arable Land Conservation, Institute of Agricultural Environment and Soil, Hainan Academy of Agricultural Sciences, Haikou, China; 2 Key Laboratory of Soil Ecosystem Health and Regulation of Fujian Provincial University, College of Resources and Environment, Fujian Agriculture and Forestry University, Fuzhou, China; 3 Hainan Key Laboratory for Sustainable Utilization of Tropical Bio-resources, College of Tropical Crops, Hainan University, Haikou, China

**Keywords:** cadmium, hyperaccumulator plants, phytoremediation, rare earth elements, rhizosphere microbes

## Abstract

Cadmium (Cd) contamination poses serious ecological and health risks, and although hyperaccumulator plants offer a sustainable phytoremediation strategy, their field performance is often constrained by low biomass and limited tolerance under metal stress. Recent studies indicate that rare earth elements (REEs), especially lanthanum (La³^+^) and cerium (Ce³^+^), can influence multiple plant and soil processes that are relevant to Cd phytoextraction. Low-dose REEs have been reported to stimulate plant growth, modulate phytohormone signaling, enhance root development, and support antioxidant responses, which may collectively improve Cd tolerance and uptake. REEs can also modify soil chemical conditions and shape rhizosphere microbial communities that contribute to Cd mobilization. In some model species, REE exposure has been associated with the induction of systemic endocytosis, a process that may provide an additional pathway for the uptake of external ions, including Cd. Here, we synthesize current evidence on how REEs interact with plant physiological processes, soil Cd speciation, and rhizosphere microbial function, and we summarize emerging insights into REE-induced systemic endocytosis. We further propose a conceptual REE–plant–soil–microbe interaction framework to integrate these regulatory pathways. Finally, we discuss key uncertainties and research needs related to mechanism verification, ecological safety, and field applicability. This review provides a foundation for evaluating the potential of REEs as regulatory agents to enhance Cd phytoremediation using hyperaccumulator plants.

## 1 Introduction and research background

The remediation of heavy metal-contaminated soils remains a significant challenge in environmental science. Globally, the accumulation of toxic metals in soils poses a major threat to agricultural productivity and food safety. For example, China’s national soil pollution survey conducted in 2014 reported that approximately 15% of agricultural soils were contaminated with heavy metals (Shi et al., 2024a). Among these pollutants, cadmium (Cd)—a non-essential and highly toxic metal—presents serious health risks through food chain transmission, leading to diseases such as Itai-itai disease (Guo et al., 2020). Phytoremediation, which employs plants to absorb, translocate, and stabilize contaminants, has attracted considerable attention due to its cost-effectiveness and environmental sustainability. In contrast, the limited metal tolerance and accumulation capacities of most conventional plant species constrain the overall effectiveness of this approach.

However, hyperaccumulator plants are capable of accumulating extraordinarily high levels of specific heavy metals in their shoots without exhibiting phytotoxic symptoms. At the soil–root interface, their accumulation efficiency depends not only on plant physiological traits but also on the bioavailable fraction of metals regulated by soil adsorption–desorption processes, particularly those mediated by soil organic matter (Baker et al., 2020; Zhong et al., 2025). For example, *Sedum alfredii* Hance, a well-known Cd hyperaccumulator, can accumulate up to 9000 mg·kg^+^¹ Cd (dry weight) in its leaves without visible toxicity symptoms (Liu et al., 2025). Hyperaccumulators offer distinct advantages in the phytoremediation of contaminated soils by efficiently translocating heavy metals to above-ground biomass, while maintaining soil structure and contributing to ecosystem stability. Nonetheless, phytoremediation using hyperaccumulators faces several limitations. Many hyperaccumulator species exhibit low biomass, slow growth rates, and narrow metal specificity, often necessitating extended timeframes to effectively reduce soil metal concentrations (Skuza et al., 2022). For instance, most hyperaccumulators target only one type of heavy metal, making them less effective for remediating soils contaminated with multiple metals (Niu et al., 2023). Consequently, improving metal uptake efficiency and accelerating the remediation process have become major research priorities. In this context, the use of exogenous regulatory agents to enhance the phytoextraction performance of hyperaccumulators has emerged as a promising strategy.

Rare earth elements (REEs), comprising the lanthanides (atomic numbers 57–71), along with scandium and yttrium, possess unique chemical properties and have been widely applied in industrial and agricultural contexts (Brioschi et al., 2013). In agricultural practice, REE-based “microfertilizers” are typically used at low application rates, and field trials in cereals and legumes have reported yield increases of up to ~10–15% under suitable conditions (Song et al., 2021; Tommasi et al., 2021). Since the late 20th century, extensive research—particularly in China—has focused on the ability of low concentrations of REEs to regulate crop physiological processes (Brioschi et al., 2013; Zhang et al., 2022b). For example, treatments with cerium chloride (CeCl_3_) or neodymium nitrate [Nd(NO_3_)_3_] increased leaf chlorophyll content by 9–40% in *Brassica napus* (Song et al., 2021), and He et al. (2020) also showed that lanthanum application (10 μM) enhanced chlorophyll concentrations in *Panicum virgatum*. Beyond photosynthesis, exogenous REEs can also modulate root exudation, improve nutrient availability, and reshape rhizosphere microbial communities, thereby promoting seedling growth in tomato (Si, 2022). In maize–black soil systems, 100 mg kg^+^¹ lanthanum increased total nitrogen, phosphorus, and potassium contents by 9–18% (Liu, 2023).

These physiological and metabolic responses provide a mechanistic basis for exploring whether REEs can also enhance metal uptake in hyperaccumulator species. Growing evidence from non-hyperaccumulator crops further indicates that REEs influence redox regulation. In *Oryza sativa*, La³^+^ treatments ranging from 0.05 to 1.5 mM significantly altered reactive oxygen species (ROS) production and antioxidant metabolism (Liu et al., 2016; Xu and Chen, 2011). Notably, exposure to 0.05 mM La³^+^ reduced ROS levels while enhancing antioxidant activity, suggesting that low-dose La³^+^ may contribute to redox homeostasis under stress conditions. Building on these physiological observations, studies in hyperaccumulator species demonstrate that REEs can also enhance heavy-metal uptake. In *Solanum nigrum* exposed to 10 mg L^+^¹ Cd, La³^+^ addition increased total fresh biomass by 42.20%, reduced leaf MDA content by 20.19%, and elevated Cd concentrations in roots, stems, leaves, and fruits by 9.22%, 21.65%, 23.95%, and 21.12%, respectively. Under 25 mg L^+^¹ Pb, foliar or hydroponic La³^+^ application increased Pb concentrations by 5.82–11.18% (roots), 8.91–17.37% (stems), 14.07–19.17% (leaves), and 21.10–27.12% (fruits). Together, these findings suggest that moderate levels of La and Ce can stimulate plant growth, improve physiological or rhizosphere conditions, and activate additional uptake pathways, thereby enhancing the accumulation efficiency of heavy metals such as Cd (Guo et al., 2024). Consequently, REE–plant interactions have attracted increasing attention as a potential strategy to improve hyperaccumulator-based phytoremediation in contaminated soils.

Compared with previous reviews that have mainly addressed REE functions in agriculture or general plant physiology, this review specifically focuses on Cd phytoremediation in hyperaccumulator systems and integrates regulatory mechanisms across plant, soil, and microbial dimensions. In particular, we highlight the emerging mechanism of REE-induced systemic endocytosis and develop an integrated REE–plant–soil–microbe interaction framework, offering a conceptual perspective not previously synthesized. We further summarize recent advances in REE regulation of plant photosynthesis, root activity, phytohormone dynamics, root exudation, soil Cd mobilization, and rhizosphere C/N metabolic processes. The review is organized around the following themes: (1) REE effects on plant physiological processes; (2) REE-mediated modulation of Cd bioavailability and soil speciation; (3) impacts on rhizosphere microbial community structure and function; (4) REE-induced systemic endocytosis; (5) an integrated REE–plant–soil–microbe regulatory model; and (6) remaining knowledge gaps and future research priorities. Through this synthesis, we aim to establish a mechanistic foundation and provide a comprehensive perspective for advancing REE-assisted, hyperaccumulator-based Cd phytoremediation.

## 2 Regulatory effects of rare earth elements on plant functions

The efficiency of heavy metal remediation by hyperaccumulator plants is largely governed by the coordinated regulation of several physiological functions, including photosynthetic efficiency, endogenous hormone levels, root development capacity, and biomass accumulation. These physiological traits not only determine the plant’s ability to absorb, transport, and accumulate heavy metal ions but also significantly influence its growth stability and remediation potential under metal stress. Therefore, optimizing the physiological status of hyperaccumulators through exogenous interventions has become a key strategy to enhance their metal extraction capacity. A growing body of evidence indicates that low concentrations of rare earth elements (REEs) can positively regulate multiple physiological processes in plants (Tao et al., 2022).

### 2.1 Promotion of plant photosynthesis by rare earth elements

Among the various REEs, lanthanum (La³^+^) has attracted particular attention for its capacity to enhance photosynthesis. Under normal growth conditions, appropriate concentrations of La have been shown to improve photosynthetic performance through multiple mechanisms. Specifically, La increases the chlorophyll and carotenoid contents in plant tissues, thereby improving light energy absorption and enhancing photosynthetic efficiency (van Doan et al., 2025). La treatment also improves pigment accumulation in leaves, elevates the net photosynthetic rate, and enhances stomatal conductance, thus promoting photosynthetic carbon assimilation (Ma et al., 2017). Moreover, La improves chloroplast ultrastructure and increases photosystem II (PSII) activity. It also facilitates photophosphorylation, boosting ATP and NADPH production, which are essential for carbon fixation (Ceccanti et al., 2023; Piccolo et al., 2021).

Under stress conditions such as Cd exposure, La exhibits notable protective effects. For example, supplementation with La under Cd stress can significantly mitigate damage to the photosynthetic apparatus, maintain higher chlorophyll levels, and sustain elevated photosynthetic rates (Huang and Zhou, 2006). Carotenoids, functioning as antioxidants, protect chlorophyll molecules from photooxidative damage and help maintain photosynthetic efficiency (Pérez-Gálvez et al., 2020). Furthermore, La stabilizes cellular membranes, reduces the accumulation of reactive oxygen species (ROS), and enhances antioxidant enzyme activities, collectively preserving photosynthetic function (Zhong and Chen, 2020). However, the physiological effects of REEs are concentration-dependent. Excessive La levels can inhibit photosynthesis and induce phytotoxicity (Jiang et al., 2023). Therefore, careful regulation of REE application concentration is essential to maximize their beneficial effects on photosynthesis.

### 2.2 Effects of rare earth elements on endogenous phytohormone levels

Phytohormones are essential signaling molecules that regulate plant growth, development, and responses to environmental stress. Their homeostasis and dynamic regulation have profound impacts on plant physiological status (Shi et al., 2024b). Recent studies have shown that REEs—particularly lanthanum (La³^+^), neodymium (Nd), and scandium (Sc)—can modulate the biosynthesis, transport, and signal transduction of phytohormones, thereby indirectly influencing plant development and stress responses.

With respect to auxins, especially indole-3-acetic acid (IAA), REEs significantly influence their biosynthesis and spatial distribution. For example, Nd treatment has been reported to increase endogenous IAA levels, promoting cell division and elongation, and thereby enhancing plant vigor (Luo et al., 2008). Transcriptomic analyses in *Arabidopsis thaliana* have shown that La exposure downregulates certain auxin-responsive genes such as IAA7, while members of the YUC gene family—YUC5 and YUC3—are downregulated, and YUC9 is upregulated, all of which are involved in root organogenesis (Grosjean et al., 2024). Furthermore, La has been shown to interfere with auxin transport by suppressing the expression of PIN proteins, which are critical for polar auxin transport. This disruption can lead to inhibited primary root growth (Chen et al., 2016). REEs also affect other growth-promoting hormones such as gibberellins (GAs). Appropriate REE dosages have been shown to enhance GA biosynthesis or signaling, thereby promoting internode elongation and seed germination *Oryza sativa* (Zhang et al., 2022b). In terms of stress responses, REEs modulate the levels of inhibitory hormones such as abscisic acid (ABA), ethylene (ET), salicylic acid (SA), and jasmonic acid (JA), thereby improving plant stress resilience. For example, terbium (Tb) treatment has been reported to increase ABA levels, enhancing tolerance to drought and salt stress (Wang et al., 2015). Gadolinium (Gd) has been found to activate JA/ET-mediated defense responses, protecting *Arabidopsis* against *Botrytis cinerea* infection (Batista-Oliveira et al., 2021). Under chromium (Cr) stress, Sc application significantly elevates ABA, SA, and JA concentrations in *Lemna minor*, enhancing antioxidant capacity and mitigating oxidative damage (Alp et al., 2023a).

Although an increasing number of studies indicate that REEs can alter endogenous phytohormone profiles, the molecular mechanisms underlying these shifts remain largely unresolved. Current evidence consistently shows that REE-induced hormonal responses are highly dose-dependent and strongly species-specific rather than following a universal regulatory pattern. For example, low concentrations of La³^+^ in cereals such as *Oryza sativa* and *Zea mays* have been associated with elevated levels of IAA, SA, GA, and CK (Alp et al., 2023b; Cui et al., 2019). By contrast, Tb³^+^ treatment in *Armoracia rusticana* suppresses auxin and GA while enhancing ABA, shifting the hormonal balance toward a stress-responsive profile (Luo et al., 2008). Beyond typical lanthanides, Sc³^+^ in *Lemna minor* provides a further example of context-specific responses: Sc increases basal IAA, GA, CK, SA, and JA levels under non-stress conditions, but partially restores these hormones when disrupted by Cr stress while suppressing excessive ABA accumulation (Alp et al., 2023a). Together, these contrasting cases suggest that REEs may act at multiple, yet poorly resolved, regulatory nodes within hormone networks—including auxin biosynthesis (YUCs), transport (PINs/AUX1), GA–DELLA signaling, and stress-hormone pathways (NCED, PP2Cs, JAZ/COI1, MYC transcription factors). However, direct biochemical or genetic evidence defining the primary molecular targets of REEs is still lacking. To move beyond correlative observations, systematic studies integrating hormone-reporter lines (e.g., DR5, ARR, JAZ/ERF reporters), phosphoproteomics, receptor-binding assays, and mutant analyses will be essential to elucidate how REEs interface with hormone signaling cascades and to determine whether these hormonal adjustments contribute directly to enhanced heavy-metal uptake in hyperaccumulator species.

### 2.3 Effects of rare earth elements on root growth and nutrient uptake

Rare earth elements (REEs) exert significant effects on root development and architecture. At the morphological level, rare earth elements (REEs) exert significant effects on root system development. Low doses of REEs consistently promote lateral root emergence and root hair elongation, thereby expanding the absorptive surface area of roots (Ma et al., 2010). Mechanistic evidence suggests that these architectural changes are closely linked to REE-induced modulation of auxin distribution and ROS–Ca²^+^ signaling. For example, La³^+^ and Ce³^+^ trigger rapid Ca²^+^ influx at the root apex, accompanied by localized ROS accumulation, which jointly regulate lateral root primordium activation through AUX1/PIN-mediated auxin transport (Thiruvengadam et al., 2024; Zhao et al., 2021). These coordinated hormonal and redox signals provide a mechanistic basis for REE-mediated modifications of root system architecture.

REEs also influence nutrient uptake by altering membrane transport processes. Physiological studies show that La³^+^ enhances plasma-membrane H^+^-ATPase activity and induces transient membrane depolarization, creating a more favorable electrochemical gradient for cation uptake (Hu et al., 2004). Single-cell ion-flux measurements (SIET) further demonstrate that Ce³^+^ increases net influxes of NO_3_^+^, NH_4_^+^, and Ca²^+^ across root epidermal cells, providing direct quantitative evidence for enhanced transmembrane ion transport. In addition, moderate REE treatments upregulate nutrient-transporter genes such as NRT1.1/NRT2.1 (nitrate), AMT1;3 (ammonium), and PHT1 phosphate transporters, supporting increased nutrient acquisition at the molecular level (Kaur et al., 2024).

Beyond membrane transport, REEs modulate cell division and root physiological activity. Low concentrations of Ce³^+^ accelerate the cell cycle and promote endoreduplication, leading to enlarged cortical cells and enhanced tissue growth (Peng and He, 2013). REEs also modify cell-wall structure by increasing pectin methylesterification and altering hemicellulose cross-linking, which improves cell-wall extensibility and root-tip penetration capacity (Wang et al., 2004). Combined with thicker root caps and elevated antioxidant enzyme activity, these cytological and structural changes contribute to higher root vigor and more efficient nutrient absorption.

Collectively, these structural, physiological, and molecular modifications significantly enhance the root’s capacity to acquire water and mineral nutrients. For instance, foliar application of moderate concentrations of lanthanum chloride (LaCl_3_) has been reported to enhance root vigor, acid phosphatase activity, and fine-root development in sweet potato (*Ipomoea batatas*) under phosphorus-deficient conditions, ultimately increasing biomass and yield. Similar improvements in nutrient uptake efficiency and the yield and quality of medicinal organs have been observed in cabbage (*Brassica oleracea*) (Jia et al., 2024). In Cd-contaminated soils, a more active and transport-efficient root system is essential for increased Cd uptake, suggesting that REE-induced enhancement of root architecture and membrane transport processes may directly contribute to improved phytoremediation performance.

### 2.4 Rare earth elements enhance plant tolerance to cadmium stress

An increasing body of evidence suggests that rare earth elements (REEs), particularly lanthanum (La³^+^) and cerium (Ce³^+^), can mitigate cadmium (Cd) toxicity in plants by enhancing stress tolerance mechanisms (Kastori et al., 2023). Exposure to Cd commonly induces the overproduction of reactive oxygen species (ROS) in plant tissues, leading to oxidative damage. REE treatments have been shown to strengthen the antioxidant defense systems in plants under Cd stress, thereby reducing ROS-mediated cellular injury. For instance, Pang et al. (2002) reported that La³^+^ application increased the activities of key antioxidant enzymes and reduced lipid peroxidation in wheat seedlings exposed to heavy metal stress. This enzymatic activity helps neutralize superoxide radicals and hydrogen peroxide generated under Cd exposure, thereby protecting cellular structures. Similarly, exogenous La³^+^ and Ce³^+^ have been found to alleviate oxidative damage in rice under environmental stress, as evidenced by increased activities of superoxide dismutase (SOD), peroxidase (POD), and catalase (CAT), alongside reduced malondialdehyde (MDA) accumulation (Adeel et al., 2019; Elbasan et al., 2020).

Another important mechanism by which REEs enhance Cd tolerance is through the regulation of metal uptake and chelation processes. At low concentrations, La³^+^ may compete with Cd²^+^ at uptake sites in roots and regulate its translocation to shoots, thereby reducing toxicity in vital plant tissues while maintaining overall Cd uptake (He et al., 2005). This effect is partly attributed to the chemical similarity between La³^+^ and Ca²^+^, as La³^+^ can competitively inhibit Cd²^+^ entry by blocking Ca²^+^ channels used by Cd. However, some studies have shown that REEs may also promote the uptake of Cd and other heavy metals under certain conditions. For example, Zong et al. (2024) reported that La treatment significantly increased Cd accumulation in both shoots and roots of lettuce by 16–30% and 16–34%, respectively, and Pb accumulation by 25–29% and 17–23%, respectively. Similarly, Ben et al. (2021) found that treatment with 30 μM La³^+^ elevated Pb concentrations in the leaves of soybean, lettuce, pak choi, and celery to 7, 17, 19, and 33 times that of the control, respectively. REE exposure has also been found to induce or upregulate the production of metal-binding chelators. He et al. (2005) demonstrated that La³^+^ and Ce³^+^ can stimulate the biosynthesis of thiol-rich ligands such as phytochelatins and metallothioneins. These molecules tightly bind Cd ions and sequester them into vacuoles, rendering the metal chemically inert. Supplementation with La has been shown to alleviate Cd-induced growth inhibition, an effect associated with increased thiol content and enhanced chelation capacity. Collectively, these findings suggest that REEs may exert dual effects on heavy metal uptake and detoxification, depending on factors such as plant species, metal type, environmental conditions, and REE concentration.

In summary, rare earth elements enhance multiple physiological functions in plants: they boost photosynthesis and antioxidant capacity, optimize hormone balance, promote root development and nutrient uptake, and improve Cd stress tolerance. For hyperaccumulator plants, these effects provide critical physiological support for efficient heavy metal absorption and accumulation in contaminated environments. As exogenous regulators, REEs offer a promising strategy to overcome growth and uptake limitations in hyperaccumulators.

## 3 Mechanisms by which rare earth elements regulate soil cd activation and speciation

To improve the extraction efficiency of heavy metals from soil by hyperaccumulator plants, it is essential not only to enhance the plants’ uptake capacity but also to increase the proportion of bioavailable heavy metal fractions in the soil—a process referred to as “activation” of heavy metals. In soils, heavy metals exist in various forms, including exchangeable, carbonate-bound, Fe-Mn oxide-bound, organically bound, and residual fractions (Shi et al., 2023). Among these, only the water-soluble and exchangeable forms are readily available for root uptake. The addition of rare earth elements (REEs) may influence the equilibrium of heavy metal speciation in the soil through multiple pathways, thereby increasing their bioavailability.

Once entering the soil solution, trivalent REEs such as La³^+^ participate in competitive surface reactions with negatively charged colloids and mineral surfaces (Wu et al., 2023). As a typical trivalent cation with strong electrostatic affinity and complexation potential, La³^+^ shows high affinity for functional groups on soil colloids, such as carboxyl (–COO^+^), phenolic hydroxyl (–OH), and phosphate (–PO_4_²^+^) groups. Compared with divalent cations, La³^+^ exhibits stronger site preference on variable-charge minerals (e.g., Fe/Al oxides and clay edges), resulting in competitive displacement of pre-adsorbed Cd²^+^ even at lower molar concentrations (Kajjumba, 2022). This competitive behavior reflects not only charge differences but also mineral-surface coordination chemistry, where La³^+^ forms more stable inner-sphere complexes, thereby weakening outer-sphere complexes formed by Cd²^+^. As a result, La³^+^ preferentially occupies adsorption sites and triggers cation-exchange reactions that release Cd²^+^ and Pb²^+^ into the soil solution. This ion-exchange process increases the proportion of exchangeable Cd and enhances its mobility and bioavailability to plants—effects that are particularly pronounced in soils rich in humus, clay minerals, and Fe–Mn oxides (Alloway, 1995).

Clay minerals such as montmorillonite and illite possess large specific surface areas and high cation exchange capacities, with abundant negatively charged sites that serve as major carriers for metal adsorption. Due to its higher valence and stronger electrostatic attraction, La³^+^ can effectively compete for these sites, disrupting the preexisting metal adsorption equilibrium and thereby reactivating adsorbed Cd and other metals (Pérez-Rodríguez and Maqueda, 2002). Moreover, La³^+^ adsorption is influenced by soil pH buffering and carbonate equilibria: in weakly acidic soils, REE salts can slightly decrease pH, enhancing the dissolution of Cd-bearing carbonates and weakening Cd sorption on Fe/Mn oxides through proton-promoted desorption. In contrast, in buffered calcareous soils where carbonate dissolution is limited, this activation effect may be attenuated (Wu et al., 2001). Additionally, the small ionic radius and relatively large hydrated radius of La³^+^ allow it to form inner-sphere complexes on mineral surfaces, including interlayer adsorption on edge structures of clay layers, which can further destabilize the adsorption of Cd and reduce its binding stability (Xie et al., 2024). These processes illustrate that Cd activation reflects a balance among competitive adsorption, mineral-surface complexation, pH buffering, and redox-sensitive oxide phases rather than simple ion exchange alone. Importantly, this exchange-driven mechanism is not limited to binary La³^+^–Cd²^+^ interactions; it may also trigger a cascade of dynamic adsorption–desorption rearrangements in multi-metal systems, indirectly altering the availability of other micronutrients. Because REEs also compete with Ca²^+^, Mg²^+^, and Zn²^+^ for similar adsorption sites, their addition can shift the broader thermodynamic equilibrium of cation distribution on colloid surfaces, thereby influencing the partitioning of both target metals (e.g., Cd) and nutrient cations. This mechanism plays a critical role in regulating metal bioavailability in the rhizosphere of hyperaccumulator plants, especially under conditions where contaminants predominantly exist in stable, non-bioavailable forms, thereby limiting remediation efficiency. The metal “activation” function of REEs thus provides a prerequisite for improving plant uptake and enhancing phytoremediation outcomes.

In addition, rare earth elements (REEs) can indirectly modulate heavy metal speciation by altering soil physicochemical properties. Studies have shown that the concentration and mobility of REEs in soil are influenced by factors such as parent material, soil texture, pH, organic matter content, and redox conditions (Mihajlovic and Rinklebe, 2018). These factors not only determine the behavior of REEs in the soil but also affect the speciation and bioavailability of other heavy metals such as Cd (Feng et al., 2023). Under anthropogenic application scenarios, the addition of REE salts (e.g., lanthanum chloride, LaCl_3_) can modify the ionic strength and pH of the soil solution. For example, if REEs lower soil pH (increase acidity), certain Cd-associated carbonates or hydroxides may dissolve, releasing free Cd²^+^ into solution. Conversely, if REEs increase soil pH, a portion of exchangeable Cd may convert into less soluble forms (Steinmann and Stille, 1997). Overall, however, in most acidic to neutral soils, the application of moderate amounts of REE salts—typically weakly acidic themselves—tends to promote Cd release rather than immobilization. Recent evidence also highlights the importance of biogeochemical nitrogen transformations in regulating Cd mobility—a dimension not yet incorporated into REE–soil interaction studies. Changes in ammonium and nitrate availability strongly influence soil pH, redox potential (Eh), and metal–ligand equilibria (Wang et al., 2024). Nitrification-driven acidification enhances the dissolution of Cd carbonates and Fe/Mn-oxide–bound Cd, whereas denitrification can alter rhizosphere redox status and promote the release of Cd²^+^ from redox-sensitive mineral phases (Zhang et al., 2022a). Studies on nitrogen fertilization further show that NO_3_^+^ can enhance Cd leaching and phytoavailability by increasing soil ionic strength and disrupting Cd–organic matter complexes (Bai et al., 2021). Given that REEs can reshape microbial communities and potentially influence N-cycling functional groups, future mechanistic models of REE-induced Cd activation should explicitly integrate nitrogen transformations and associated redox dynamics.

Moreover, REEs can react with strongly coordinating soil constituents such as phosphate and organic matter, potentially sequestering ligands that would otherwise bind Cd. A typical example is the strong affinity between La³^+^ and phosphate, leading to the formation of insoluble lanthanum phosphate complexes and thus reducing the available phosphorus in soil. In this process, phosphate ions that would otherwise precipitate or complex with Cd are sequestered by La³^+^, indirectly increasing the proportion of free Cd in the soil solution (Cao et al., 2001).

Third, the physiological effects of REEs on plant roots may feed back to influence the speciation and mobility of heavy metals in the rhizosphere. Many hyperaccumulator plants release organic acids (e.g., oxalic acid, malic acid) and other chelating compounds into the rhizosphere to mobilize heavy metals (Osmolovskaya et al., 2018). While REEs have been shown to enhance root vigor, metabolic activity, and membrane permeability, direct evidence demonstrating that REEs quantitatively increase organic-acid exudation remains limited. Existing findings mainly support indirect mechanistic links—for example, increased root activity and enhanced redox/energy status under REE treatment, which could plausibly facilitate solute release. Given this context, the notion that REEs stimulate root exudation should be interpreted as a mechanistic hypothesis rather than a confirmed physiological response. Nevertheless, if REEs enhance root metabolic activity and membrane transport, the resulting shifts in exudate quantity or composition could plausibly chelate sparingly soluble Cd forms, thereby increasing their mobility. Supporting this conceptual pathway, isotope tracing in *S. alfredii* has shown that root-derived exudates can restructure rhizosphere microbial communities and promote Cd mobilization and uptake (Li et al., 2010), suggesting how REE-induced changes in root physiology might indirectly influence Cd activation.

In summary, REEs regulate soil Cd speciation primarily through established chemical processes—including competitive adsorption, inner-sphere complexation, and pH-driven dissolution of carbonate and Fe/Mn-oxide phases—and through potential biological pathways such as enhanced root activity and rhizosphere restructuring. These interacting mechanisms likely contribute to the increased transfer of Cd from soil to roots. To establish causal mechanistic links, future research should incorporate quantitative exudate-flux measurements (e.g., microdialysis, exudate trapping), metabolomic profiling, and isotope-labeled metal-flux assays to verify the biological contributions to REE-mediated Cd activation.

## 4 Effects of rare earth elements on rhizosphere microbial community structure and carbon/nitrogen metabolism

The rhizosphere is the most dynamic interface for interactions among plants, soil, and microorganisms, often referred to as the convergence zone of the “microbial sphere” and the “rhizosphere food web” (Berendsen et al., 2012). In heavy metal-contaminated environments, rhizosphere microbial communities play a vital role in plant survival and remediation efficiency. Certain beneficial microbes can promote plant growth and enhance heavy metal uptake by fixing nitrogen, solubilizing phosphorus, producing growth-promoting substances, or secreting metal chelators. Conversely, metal stress can significantly alter microbial community composition and metabolic function (Barra Caracciolo and Terenzi, 2021). Microorganisms themselves also regulate heavy metal bioavailability in soils through mechanisms such as secretion of organic acids, siderophores, and efflux pumps, which alter metal speciation and mobility, ultimately influencing the concentration of bioavailable metal ions for plant uptake (Etesami, 2018). Beyond ligand-driven metal mobilization, rhizosphere microorganisms also mediate redox-dependent transformations that strongly regulate Cd mobility. In partially saturated or micro-anaerobic microsites, Fe(III)- and Mn(IV)-reducing bacteria can dissolve Fe/Mn oxides—key sorbents for Cd—thereby releasing previously immobilized Cd²^+^ into the soil solution (Huang et al., 2023). Conversely, nitrate-dependent Fe(II)/Mn(II) oxidation or coupled nitrification–denitrification can regenerate reactive oxide surfaces that either adsorb or release Cd depending on pH and Eh (Hu et al., 2025). These microbial redox loops, well-documented in As and Mn cycles of flooded soils, fundamentally reshape metal–ligand equilibria and should be integrated into REE–soil interaction frameworks. For example, nitrate reduction–linked Mn(II) oxidation has been shown to suppress As(III) mobilization and N_2_O emissions in flooded paddy soils (Wang et al., 2024), demonstrating that biogeochemical N transformations can directly modify redox-sensitive metal dynamics. When rare earth elements (REEs) are introduced into phytoremediation systems, they can similarly influence the structure and function of rhizosphere microbial communities (Jiang et al., 2024). These effects may arise from the direct physiological toxicity or stimulatory effects of REEs on microbes, or indirectly via changes in plant metabolism, root exudate composition, and the physicochemical conditions of the rhizosphere.

From the perspective of microbial community structure, the presence of REEs can alter microbial diversity and the dominance of specific taxa in the rhizosphere. For example, studies conducted in paddy soils near rare earth mining areas showed that high REE concentrations reduced the α-diversity of soil and endophytic bacterial communities, leading to decreased species richness and evenness (Zhang et al., 2022b). This suggests that REEs may suppress sensitive microbial taxa and shift the community structure toward a few REE-tolerant groups. At the same time, certain functional microbes may be selectively enriched under REE stress. In the aforementioned study, REE-contaminated paddy fields were found to harbor enriched populations of *Burkholderia*, *Bacillus*, *Buttiauxella*, *Acinetobacter*, and *Bradyrhizobium* in both the rhizosphere and plant tissues. These dominant taxa possess multiple beneficial traits, including the ability to degrade pollutants from REE mining activities, mitigate combined REE–ammonium toxicity, and exhibit nitrogen fixation and REE tolerance (Zhang et al., 2025, 2016). Moreover, in response to nutrient imbalances caused by REEs—such as reductions in available phosphorus—microbial communities may adapt by enriching phosphorus-solubilizing genera like *Pantoea* to compensate for phosphorus deficiency (Jin et al., 2019). These findings suggest that REEs reshape rhizosphere microbial communities by altering soil chemistry and exerting selective pressure, driving microbial succession toward structures better adapted to the new environment and, in some cases, beneficial to the host plant. Furthermore, rhizosphere microbes play a crucial role in regulating heavy metal speciation and bioavailability in soil. Some functional bacteria secrete organic acids (e.g., oxalic and citric acids) and metal chelators (e.g., siderophores) or modify the local pH and redox conditions, thereby promoting the desorption or solubilization of otherwise immobile metals. For example, organic acid-producing bacteria can locally acidify the rhizosphere, enhancing the release of Cd²^+^ from soil colloids (Shi et al., 2024c). Phosphate-solubilizing and nitrogen-fixing bacteria can also indirectly alter the coordination environment of metal ions through the release of cofactors and metabolites (Oleńska et al., 2020). In addition, some microbes possess metal transport or redox capabilities, converting Cd and other metals into more bioavailable or less toxic forms (Etesami, 2018). The enrichment or functional activation of such microbial groups by REEs may enhance plant uptake of Cd and other heavy metals. Therefore, the REE-induced restructuring of microbial communities not only affects the plant growth environment but also plays an integral role in regulating heavy metal mobility and bioavailability. This provides microecological support for improving phytoremediation efficiency.

Beyond altering community composition, rare earth elements (REEs) exert more profound impacts on the functional activities of rhizosphere microorganisms, particularly on microbial-mediated carbon (C) and nitrogen (N) cycling processes. Studies combining metagenomic analysis and soil enzyme assays have shown that increasing concentrations of La and Ce in soil significantly enhance microbial activities associated with carbon degradation and nitrogen transformation (Song et al., 2025). In a controlled soil incubation experiment, high doses of Ce (0.16–0.32 mmol·kg^+^¹) increased soil CO_2_ emission rates by approximately 40–59%, and N_2_O emission rates by 255–609%. Similarly, comparable concentrations of La elevated CO_2_ production by 39–47% and N_2_O production by 105–187%. These results indicate that elevated REE levels strongly stimulate microbial respiration and denitrification. In addition to changes in gene abundance, REE exposure has been shown to alter redox potential (Eh) in the rhizosphere by stimulating heterotrophic respiration and oxygen consumption, as evidenced by significantly elevated CO_2_ and N_2_O fluxes under La and Ce treatments (Song et al., 2025). The resulting shift toward lower Eh creates micro-anaerobic zones that favor denitrification and dissimilatory nitrate reduction (Lovley, 1991). These anaerobic conditions also promote the reductive dissolution of Fe/Mn (oxyhydr)oxides, a process known to liberate sorbed Cd into the soil solution (Grybos et al., 2007). Enzyme activity assays in REE-treated soils further support this trend, showing increased activities of nitrate reductase, nitrite reductase, and dehydrogenases consistent with intensified electron-transfer pathways during N turnover (El-Ramady, 2011). Taken together, these findings demonstrate that REEs not only restructure microbial communities but also shift the rhizosphere toward a more reducing and metabolically active state, which can facilitate Cd mobilization and enhance plant uptake.

Metagenomic data revealed a significant increase in the abundance of functional genes involved in carbon degradation under REE treatment, including those encoding enzymes for the breakdown of cellulose, hemicellulose, lignin, chitin, and peptidoglycan. Genes related to nitrogen cycling were also upregulated, such as those involved in organic nitrogen mineralization and assimilation (e.g., asnB encoding asparagine synthetase and gdh encoding glutamate dehydrogenase), and key enzymes in denitrification pathways, including nitrate reductase (nar) and nitrous oxide reductase (nosZ), which showed concentration-dependent enrichment under La and Ce exposure. These findings collectively suggest that REEs accelerate microbial-mediated carbon mineralization and nitrogen turnover in soils (Schwaner and Kelly, 2019). Such stimulation likely results from the selective proliferation of microbial taxa specialized in organic matter decomposition and nitrogen transformation. For instance, cellulolytic bacteria, ammonia-oxidizing archaea, and denitrifiers may gain a competitive advantage under REE exposure, thereby enhancing the overall carbon and nitrogen cycling functionality of the community (Krause et al., 2017; Nemergut et al., 2008).

In the rhizosphere of hyperaccumulator plants, enhanced microbial carbon and nitrogen metabolism has multiple implications. On the one hand, accelerated organic matter decomposition and mineralization release more nutrients—such as inorganic nitrogen—into the soil, improving soil fertility and promoting plant growth (McLatchey and Reddy, 1998). Increased nutrient availability helps hyperaccumulator plants overcome nutrient limitations under metal stress and supports the development of vigorous shoots and roots for pollutant uptake (Zeng et al., 2024). On the other hand, by-products of nitrogen cycling processes, such as N_2_O produced via denitrification, may alter rhizosphere chemistry by consuming NO_3_^+^ and creating localized anaerobic conditions. These changes can influence redox potential and ionic equilibria in the root zone, thereby affecting the speciation and uptake dynamics of heavy metals (Lu et al., 2022; Maurer et al., 2021). Nevertheless, as long as REE concentrations remain within an appropriate range, their stimulatory effects on microbial metabolism are likely to be beneficial—enhancing nutrient supply and promoting the activity of plant growth-promoting or metal-mobilizing microbial populations. It is worth noting that beneficial rhizosphere microbes also contribute to heavy metal tolerance and uptake by producing plant growth-promoting substances (PGPR products), fixing atmospheric nitrogen, and synthesizing metal chelators (Hayat et al., 2010). REE-induced microbial community restructuring may reinforce these functions. For instance, studies on *S. alfredii* have shown that its hyperaccumulating ecotype selectively recruits rhizosphere taxa such as *Rhodanobacter*, *Nocardioides*, and *Burkholderia*, which are believed to facilitate Cd mobilization and uptake (Hu et al., 2024). If REE treatments further stimulate the proliferation or metabolic activity of such beneficial microbes, this could synergistically enhance phytoremediation performance.

In summary, REEs profoundly influence rhizosphere microbial ecology by reshaping community structure to favor REE-tolerant, functionally specialized taxa and by enhancing microbial-mediated carbon degradation and nitrogen cycling. These changes, in conjunction with plant–microbe interactions, can significantly improve the growth and metal extraction capabilities of hyperaccumulator plants. However, attention should also be paid to potential negative effects such as reduced microbial diversity and context-dependent outcomes across different plant–soil systems. With the application of metagenomics, metatranscriptomics, and other advanced molecular tools, we are progressively uncovering the functional evolution of rhizosphere microbial communities under REE exposure. This knowledge will be instrumental in advancing our mechanistic understanding of REE–plant–microbe synergy in heavy metal phytoremediation.

## 5 Rare earth element-induced systemic endocytosis enhances heavy metal uptake

**Figure 1:**
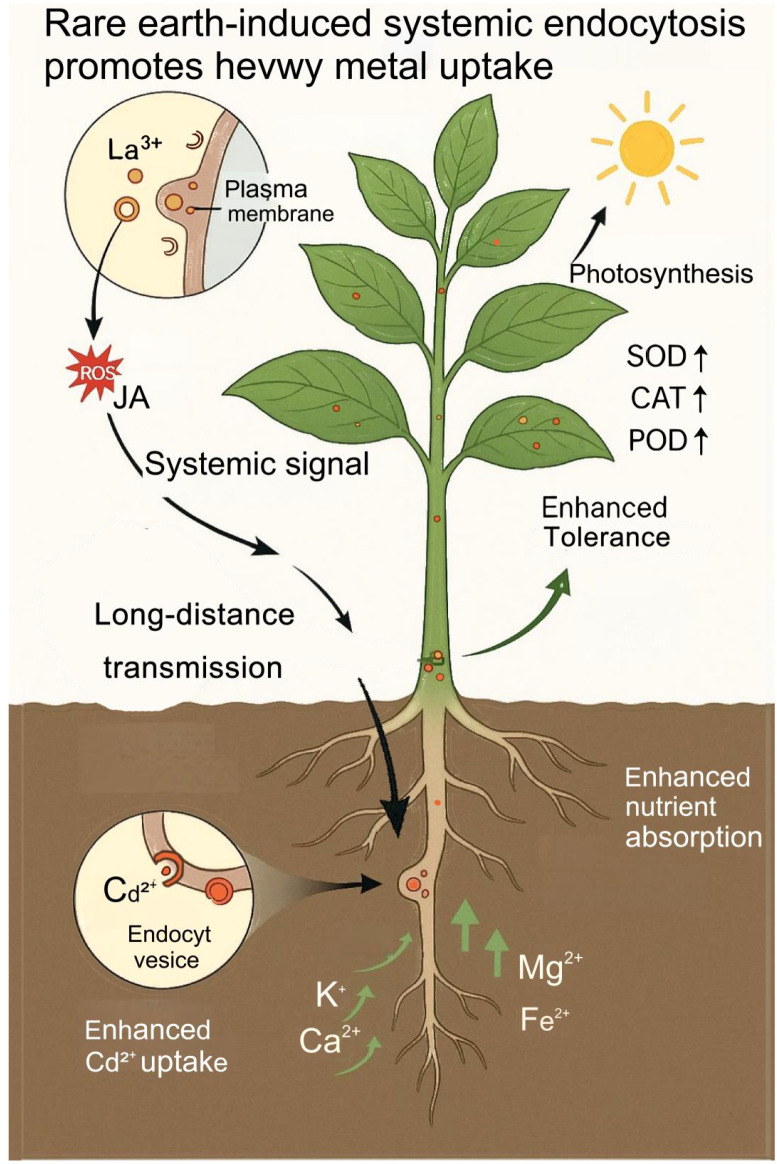
Schematic illustration of rare earth element (La³^+^)-induced systemic endocytosis enhancing heavy metal uptake in plants.

Rare earth elements (REEs), particularly lanthanum (La³^+^), have been reported to induce a distinctive form of endocytosis in plant cells. Studies using radiolabeled La and high-resolution microscopy show that foliar-applied La³^+^ binds to the plasma membrane, forms nanoscale aggregates, and triggers membrane invagination and vesicle formation—an effect not observed with most other metal ions (Liu et al., 2024). This phenomenon, referred to as systemic endocytosis, describes a whole-plant response in which La perception in the leaf activates vesicle formation in distant tissues such as roots. Mechanistically, current evidence points to a ROS–jasmonic acid (JA) signaling cascade: La stimulation in the shoot enhances ROS production, elevates JA levels, and activates endocytic activity throughout the plant (Šamaj et al., 2005). In *A. thaliana*, blocking ROS production or mutating AtrbohD abolishes the systemic endocytic response (Grosjean et al., 2024), supporting a shoot-to-root signaling mechanism mediated by redox and hormonal cues. As a result, La application increases vesicle-mediated transport capacity in root tissues, potentially modifying root physiology and mineral uptake characteristics (see **Figure 1**).

Lanthanum-induced systemic endocytosis is consistently associated with enhanced heavy-metal uptake across several hyperaccumulator species. Unlike transporter-mediated uptake, endocytosis enables bulk, partially non-selective internalization of solutes or particles from the apoplast (Chen et al., 2024). In *S. alfredii*, foliar La³^+^ application triggers systemic endocytosis in roots and substantially increases Cd²^+^ absorption and translocation to shoots (Liu et al., 2024). In *S. nigrum*, La treatment similarly enhances root endocytosis and elevates Cd and Pb fluxes by 15.6–75.9%, accompanied by higher bioconcentration and translocation factors (Guo et al., 2024). These observations suggest that systemic endocytosis enhances the efficiency of metal acquisition and may operate alongside conventional transporter pathways.

In addition to augmenting metal uptake, La-induced endocytosis is linked to broader physiological improvements under metal stress. La treatment enhances the accumulation of essential nutrients (e.g., K, Ca, Mg, Fe), reinforces ROS-scavenging capacity through increased SOD, POD, and CAT activities, and improves chlorophyll content and photosynthetic efficiency (Guo et al., 2024). The resulting improvements in nutrient status, redox homeostasis, and photosynthesis support biomass accumulation and maintain growth under contaminated conditions (Liu et al., 2024). Together, these responses indicate that La³^+^ functions both as a signaling cue triggering endocytic activity and as a physiological stimulant enhancing plant resilience.

Although current research is centered primarily on Cd, similar endocytic responses have been documented for Pb in *S. nigrum* (Guo et al., 2024). Given the partially non-selective nature of vesicle-mediated uptake, it is plausible that other cations (e.g., Zn²^+^, Cu²^+^) or even non-metal contaminants may also enter via this pathway. Supporting this idea, La foliar treatment facilitates the uptake of both inorganic and organic arsenic species in *S. nigrum* (He et al., 2023) and enhances the co-uptake of Cd and polystyrene nanoplastics in *S. alfredii* (Liu et al., 2024). These observations indicate that La-induced endocytosis can broaden the spectrum of contaminants accessible to plants beyond classical transporter-mediated pathways.

Nevertheless, the current evidence is primarily phenomenological—derived from imaging, ROS/JA signaling analyses, and tracer uptake—and offers limited insight into the biophysical constraints of the process, such as vesicle formation kinetics, vesicle size distribution, ATP consumption, or the relative contribution of endocytosis compared with canonical transporters (e.g., ZIP, NRAMP, HMA families). Moreover, species-specific differences are likely: while hyperaccumulators such as *S. alfredii* and *S. nigrum* exhibit strong systemic responses, it remains unclear whether non-hyperaccumulators possess similar regulatory capacity or signaling sensitivity. Future work integrating live-cell quantitative imaging, energy-dependence assays, pharmacological inhibition, and cross-species comparative analyses will be essential to determine the mechanistic limits, energetic costs, and ecological relevance of systemic endocytosis.

Overall, La-induced systemic endocytosis represents a promising and mechanistically distinct strategy for enhancing plant uptake of Cd, Pb, and potentially a broader range of contaminants. Its practical application will require careful control of foliar dosage, as excessive La can disrupt nutrient balance or cause phytotoxic effects. When properly optimized, however, this mechanism offers a powerful avenue for improving phytoremediation efficiency in multi-metal or co-contaminated soils.

## 6 Integrated network model and regulatory strategy

**Figure 2:**
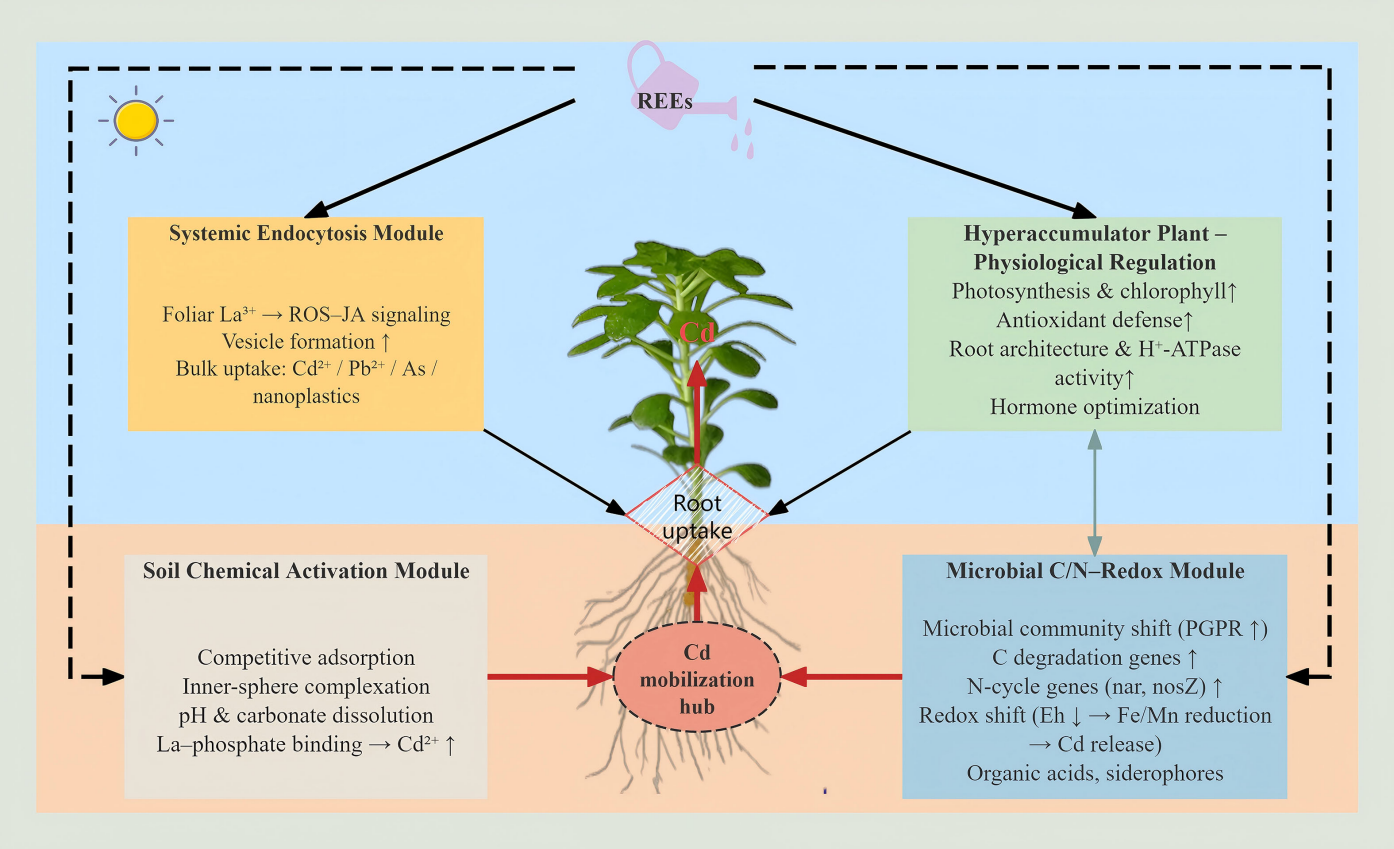
Conceptual REE–plant–soil–microbe interaction network. Arrows represent qualitative directions of influence rather than quantitative fluxes.

Based on the mechanisms described above, we propose an integrated network model linking rare earth elements (REEs), hyperaccumulator plants, soil processes, and rhizosphere microorganisms to explain how REEs enhance Cd phytoextraction (**Figure 2**). In this conceptual framework, REEs act as exogenous regulators that simultaneously modulate plant physiology, soil Cd speciation, microbial carbon/nitrogen metabolism, and vesicle-mediated uptake, thereby coordinating multiple processes that together increase Cd transfer from soil to shoots.

At the plant level, REEs enhance photosynthetic performance, strengthen antioxidant defense systems, and optimize hormone balance, which collectively support biomass production and Cd tolerance (Section 2). REEs also reshape root system architecture and membrane transport capacity—via auxin/ROS–Ca²^+^ signaling, H^+^-ATPase activation, and upregulation of nutrient transporters—thereby increasing the effective absorbing surface and ion-uptake efficiency of roots (Section 2). In Cd-contaminated soils, these changes provide the physiological and physical basis for higher Cd influx and long-distance transport.

At the soil–chemical level, REEs regulate Cd activation through competitive adsorption, inner-sphere complexation, and pH-buffer–dependent shifts in carbonate and Fe/Mn-oxide equilibria (Section 3). Trivalent La³^+^ and related REEs can outcompete Cd²^+^ for key adsorption sites on Fe/Al oxides and clay edges, displacing sorbed Cd into the soil solution and increasing the exchangeable fraction. REE-induced pH changes, coupled with intrinsic buffering and carbonate dissolution, further influence the dissolution of Cd-bearing carbonates and the stability of Cd bound to Fe/Mn (oxyhydr)oxides. In addition, strong REE–phosphate complexation can sequester phosphate ligands that would otherwise co-precipitate or complex with Cd, indirectly increasing free Cd²^+^ in solution. These processes together determine how REE inputs shift the thermodynamic distribution of Cd among solid-phase pools and the soil solution.

At the microbial and biogeochemical level, REEs reshape rhizosphere community structure and stimulate functionally specialized taxa involved in carbon degradation, nitrogen cycling, and metal mobilization (Section 4). Metagenomic and enzyme-activity data show that REE exposure can enrich plant growth–promoting rhizobacteria, increase the abundance of genes related to organic matter decomposition, N transformation (e.g., mineralization, nitrification, denitrification), and upregulate key enzymes such as nitrate reductase, nitrous oxide reductase, and dehydrogenases. These functional shifts accelerate soil carbon mineralization and nitrogen turnover, and drive redox changes in the rhizosphere (e.g., micro-anaerobic zones under elevated respiration). In turn, such redox dynamics can promote the reductive dissolution of Fe/Mn (oxyhydr) oxides and the release of sorbed Cd, while nitrate-driven processes modulate the regeneration of reactive oxide surfaces and the balance between Cd sorption and desorption. Thus, REE-induced reorganization of microbial C/N cycling provides a mechanistic link between nitrogen biogeochemistry, redox potential (Eh), and Cd mobility.

A biological and biogeochemical feedback loop further connects these components. REE-enhanced plant growth and root activity increase the flux of root exudates (e.g., sugars, organic acids, amino acids, signaling molecules), which serve as substrates and signals for rhizosphere microbes. In response, enriched functional microbes produce organic acids, siderophores, and phytohormones, acidify microenvironments, chelate or transform Cd, and promote nutrient availability, thereby feeding back to support plant growth and Cd uptake. In parallel, microbial N cycling and associated N_2_O/CO_2_ emissions adjust local Eh and NO_3_^+^/NH_4_^+^ balance, indirectly altering Cd speciation and transport in the root zone. These plant–microbe–soil feedbacks form a dynamic “biological pump” that continuously mobilizes Cd toward the root–soil interface.

Superimposed on these processes, REE-induced systemic endocytosis provides a qualitatively distinct, vesicle-mediated pathway for contaminant uptake (Section 5). Foliar La³^+^ application triggers a ROS–JA-dependent signaling cascade that enhances endocytic activity in roots, increasing vesicle formation and facilitating bulk internalization of apoplastic Cd²^+^ (and, in some cases, Pb and As species, as well as nanoplastics). This vesicle-mediated pathway operates alongside transporter-based uptake (e.g., ZIP, NRAMP, HMA transporters) and may be particularly important for hyperaccumulators with strong systemic endocytic responses, such as *S. alfredii* and *S. nigrum*.

It is important to emphasize that **Figure 2** is a qualitative, hypothesis-driven model rather than a quantitative mass-balance diagram. The arrows represent the direction of influence among processes (e.g., “REEs → enhanced microbial denitrification → Fe/Mn-oxide reduction → Cd release”), but do not imply measured flux magnitudes or rate constants. Likewise, only some of the feedbacks (e.g., REE effects on root architecture, microbial C/N genes, and Cd speciation) are currently supported by quantitative data, whereas others (such as REE-induced shifts in exudation patterns or the relative contribution of systemic endocytosis vs. transporter uptake) remain to be experimentally constrained.

Overall, this conceptual REE–plant–soil–microbe network highlights four key regulatory dimensions by which REEs may enhance Cd phytoextraction: (i) optimization of plant physiological status and root architecture; (ii) chemical activation of soil-bound Cd via competitive sorption and pH/carbonate/oxide equilibria; (iii) restructuring of rhizosphere microbial communities and C/N–redox dynamics that control Cd mobility; and (iv) activation of systemic endocytosis as a complementary uptake route. Future research combining isotope-based flux tracing, omics-guided functional assays, *in situ* Eh/pH monitoring, and quantitative endocytic measurements will be essential to parameterize this framework and evaluate the relative contribution of each pathway across different plant–soil systems.

## 7 Challenges and future perspectives

Although rare earth elements (REEs) show clear potential for enhancing phytoremediation through physiological stimulation, soil chemical regulation, and rhizosphere restructuring, their practical application remains constrained by several ecological, agronomic, and economic limitations that must be critically addressed before field deployment can be considered.

A key challenge is the incomplete mechanistic resolution. Current insights—particularly concerning REE-induced signaling, transporter interactions, and systemic endocytosis—are derived from limited hyperaccumulator models and controlled environments. How these mechanisms operate across diverse plant species, soil types, and fluctuating field conditions remains largely unknown. Moreover, REE responses are strongly element- and formulation-specific, yet systematic comparisons among La, Ce, Nd, or nano-forms are scarce. Equally critical are the environmental persistence and ecological risks. REEs are chemically stable and prone to long-term accumulation in soils. Their chronic impacts on soil fauna, microbial networks, nutrient cycling, or food-chain transfer remain insufficiently characterized, raising legitimate concerns about ecological thresholds and potential secondary pollution. At the field scale, soil heterogeneity and agronomic variability pose additional constraints. The behavior of REEs is highly sensitive to pH, clay content, organic matter, redox oscillations, and fertilizer regimes. Such variability may dampen, modify, or even reverse REE-mediated activation effects documented in hydroponic or pot experiments. Meanwhile, cost–benefit considerations and application logistics—dosage control, timing, foliar versus soil delivery, compatibility with cropping systems—remain unresolved, limiting the agronomic feasibility of REE-based interventions.

Future research should therefore prioritize: (1) Ecological safety thresholds and dose–response frameworks, including long-term monitoring of REE persistence, mobility, and bioaccumulation; (2) Mechanistic refinement using multi-omics, quantitative imaging, and cross-species comparative analyses to clarify signaling, transporter interactions, and energy requirements of systemic endocytosis; (3) Integration of redox-driven microbial biogeochemistry into REE–soil models, given the strong influence of N, Mn, Fe, and As cycles on metal mobility; (4) Comparative evaluation of different REEs and formulations to identify optimal options for specific contaminants and soils; (5) Development of agronomically viable application strategies, such as micro-dosing, slow-release REE carriers, or synergy with PGPR and soil amendments; (6) Multi-season, field-scale demonstrations to validate performance under real environmental variability and assess economic feasibility.

In summary, REE-assisted phytoremediation offers a compelling conceptual framework for enhancing plant performance and heavy-metal extraction, yet its practical value will depend on resolving safety concerns, ensuring economic viability, and establishing mechanistic robustness across diverse field conditions. A coordinated research effort—linking mechanistic biology, soil biogeochemistry, agronomy, and environmental risk assessment—will be essential to determine whether REE-based strategies can evolve from experimental promise to sustainable, scalable solutions for contaminated-soil restoration.

## Data Availability

The original contributions presented in the study are included in the article/supplementary material. Further inquiries can be directed to the corresponding author.
